# Usefulness of the Phalen Test and the Tinel Sign in the Prognosis and the Impact on Quality of Life of Patients with Carpal Tunnel Syndrome Undergoing Classical Open Carpal Tunnel Release*

**DOI:** 10.1055/s-0044-1779318

**Published:** 2024-03-21

**Authors:** Isabelle Spirandelli Pimentel, Victor Spirandelli Pimentel, Flavio Faloppa, João Carlos Belloti, Marcel Jun Sugawara Tamaoki, Benedito Felipe Rabay Pimentel

**Affiliations:** 1 Faculdade de Medicina de Petrópolis, Centro Universitário Arthur Sá Earp Neto (Unifase), Petrópolis, RJ, Brasil; 2 Departamento de Ortopedia e Traumatologia, Irmandade da Santa Casa de Misericórdia de São Paulo (ISCMSP), São Paulo, SP, Brasil; 3 Departamento de Ortopedia, Escola Paulista de Medicina, Universidade Federal de São Paulo (Unifesp), São Paulo, SP, Brasil; 4 Serviço de Ortopedia e Traumatologia, Hospital Municipal Universitário de Taubaté (HMUT), Taubaté, SP, Brasil; 5 Hospital Regional do Vale do Paraíba (HRVP), Complexo Hospitalar do Vale do Paraíba, Faculdade de Medicina, Universidade de Taubaté (Unitau), Taubaté, SP, Brasil

**Keywords:** carpal tunnel syndrome, prognostic factor, quality of life, clinical course

## Abstract

**Objective:** To evaluate the usefulness of the Phalen test and the Tinel sign in the prognosis and the impact on quality of life in the clinical course of patients with carpal tunnel syndrome undergoing surgical treatment through the traditional open approach.

**Methods:** The present is a cohort study on prognosis. We included 115 patients with high probability of receiving a clinical diagnosis of carpal tunnel syndrome with indication for surgical treatment. All patients underwent the Phalen test and Tinel sign and answered the Boston Carpal Tunnel Questionnaire before and after the surgical treatment.

**Results:** The estimates for the probability of the time until remission of the Phalen test at 2, 4 and 16 weeks postoperatively were of 3.54% (95% confidence interval [95%CI]: 1.16%–8.17%), 0.88% (95%CI: 0.08%–4.38%) and 0.88% (95%CI: 0.08% to 4.38%) respectively, and, for the Tinel sign, they were of 12.39% (95%CI: 7.13%–19.18% ), 4.42% (95%CI : 1.65%–9.36%) and 2.65% (95%CI : 0.70%–6.94%) respectively. There was a reduction in the postoperative score on the Boston Carpal Tunnel Questionnaire of 1.8 points for symptom severity ( *p* < 0.001) and of 1.6 points for functional status ( *p* < 0.001).

**Conclusion:** Phalen test remission was earlier than that of the Tinel sign, but, when performed as of the second postoperative week, they were prognostic factors favorable to the clinical course, with improved quality of life.

## Introduction

Carpal tunnel syndrome (CTS) is characterized by compression of the median nerve in the wrist. 1 In the routine clinical practice, we traditionally use the results of the Phalen test and Tinel sign as the most representative clinical findings in the clinical diagnosis of CTS. 2 3 4 Nevertheless, these provocative clinical tests go unnoticed by patients; however, they can be invaluable in assessing the clinical progression of individuals with CTS. These tests have relevance not only in the clinical diagnosis but also in the evaluation of treatment effectiveness and prognosis. 5 6 Quality prognostic studies lead to a better understanding of disease progression, better targeting of effective treatments that mitigate progression, and provide more reliable information about the risk of a negative outcome to be communicated to patients. 6 As an evidence-based clinical reasoning model, the present study proposes to evaluate the usefulness of the Phalen test and the Tinel sign in the prognosis and the impact on quality of life in the clinical course of patients with a high probability of a clinical diagnosis of CTS who underwent the traditional open surgical treatment.

## Materials and Methods

**Fig. 1:**
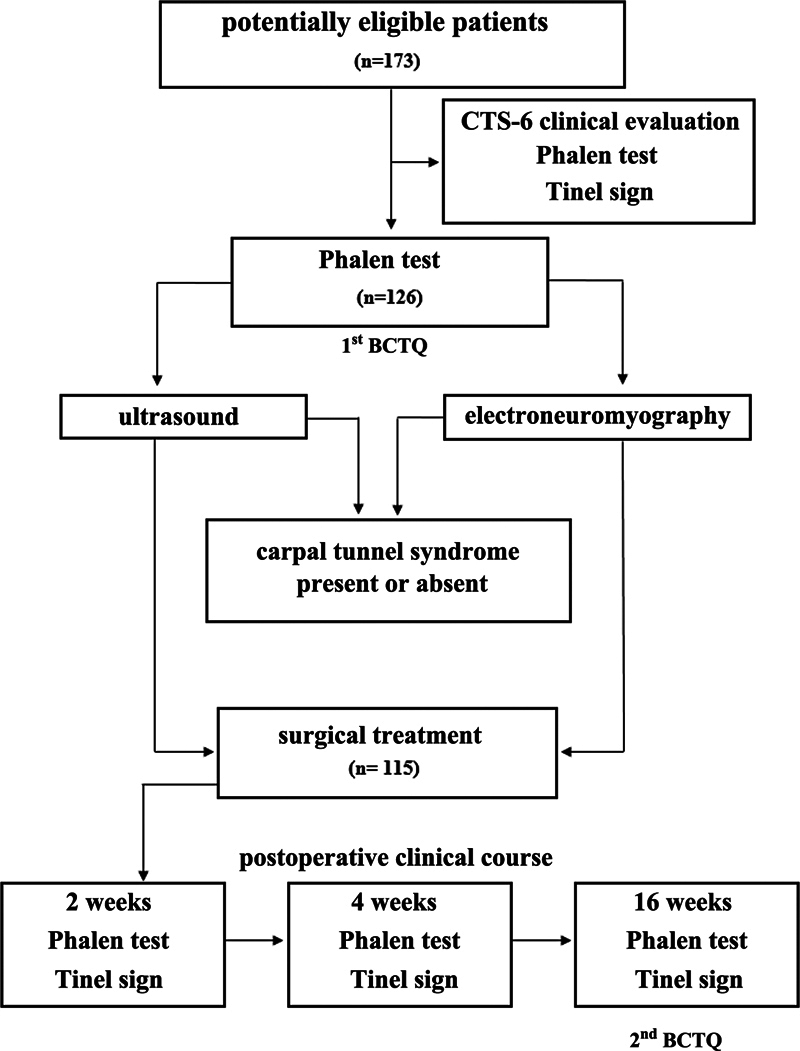
Study flowchart. Abbreviations: CTS-6, Six-item CTS Symptoms Scale; 7 BCTQ, Boston Carpal Tunnel Questionnaire. 8

The present is a single-center, primary, longitudinal, prospective cohort study on prognosis approved by the Ethics in Research Committee of our institution. We included 115 female patients aged between 40 and 80 years, who agreed and signed the free and informed consent form. The patients had undergone the conservative treatment without effective clinical improvement and with an indication for surgical treatment, and they presented a score ≥ 12 points on the 6-item CTS Symptoms Scale (CTS-6), which indicates the probability of a clinical diagnosis of CTS. 7 The 6 diagnostic criteria of this instrument with their respective scores are as follows: paresthesia (3.5), nocturnal paresthesia (4.0), hypotrophy and/or atrophy of the thenar muscles (5.0), Tinel sign (4.0) , Phalen test (5.0), and static 2-point discrimination test (5.0). 7 We excluded patients with cervical radiculopathy, other compressive syndromes of the upper limbs, polyneuropathy, history of surgical release of the carpal tunnel, and sequelae of wrist fractures. We recruited 10% more than the total of eligible patients required to cover possible losses or exclusions during the study. All eligible patients filled out the Boston Carpal Tunnel Questionnaire (BCTQ) in the preoperative period and at the end of postoperative follow-up. The BCTQ is a specific self-administered CTS questionnaire translated and validated for the Portuguese language, which assesses two scales: one for symptom severity and another for functional status. 8 As routine clinical practice, patients underwent wrist ultrasound (US) and electroneuromyography (ENMG). Then, they underwent the surgical treatment, which was performed consecutively by the same hand surgeon in the outpatient surgical center of our institution's hospital. The surgical technique used was traditional open carpal tunnel release. 9 After the surgical treatment, the patients were submitted to outpatient postoperative follow-up for 16 weeks. The Phalen test and Tinel sign were performed once preoperatively, as part of the clinical diagnosis of CTS, and at 2, 4, and 16 weeks during the postoperative clinical course. In the Phalen test, the patient rests on the examination table with the elbow positioned at 90° of flexion and the wrist positioned in maximum passive flexion of the affected hand. 10 11 The onset of paresthesia in the territory of the median nerve distribution in the hand after 30 to 60 seconds indicates a positive test. In the Tinel sign, digital percussion is performed over the region of the distal flexion crease of the wrist along the path of the affected median nerve in the hand. 11 The sensation of “shock” or discomfort at the site of percussion or radiating to the distribution territory of the median nerve in the affected hand indicates a positive test. Fig. 1 briefly shows each stage of the present study.

As the primary outcome of the present study, the estimated probability of time until remission at each assessment point and the average time until remission of each type of clinical test was estimated by Kaplan-Meier curve analysis. As a secondary outcome, the changes in BCTQ scores were estimated using analysis of variance (ANOVA) and the Kolmogorov-Smirnov test. For all statistical tests, a significance level of 5% was adopted. The statistical analyzes were performed using the following software: IBM SPSS Statistics for Windows (IBM Corp., Armonk, NY, United States), version 20.0, and Stata (StataCorp LLC, College Station, TX, United States), version 17. 12

## Results

The mean age of the 115 patients was of 52.9 (standard deviation [SD] = ± 9.1) years, with a minimum 40 years and a maximum of 79 years. Regarding the duration of the disease, the mean was of 4 (SD = ± 3.2) years), with a minimum of 1 year and a maximum of 20 years. The median was of 3 years of illness. Postoperative complications were observed in 5 patients (4.3%): 1 patient developed complex regional pain syndrome, another one presented dehiscence of the surgical scar due to a superficial infection, 1 patient developed a hypertrophic and painful scar, and 2 other patients developed pain in the bone pillar.

Table 1 shows that the incidence of remission of Phalen test 2 weeks postoperatively was higher than that of the Tinel sign ( *p* = 0.006). There were no differences in the incidence of remission at the other evaluation moments of the two clinical tests.

**Table 1:** Incidences of remission by type of test and moments of evaluation

Preoperative period	Phalen test		Tinel sign		*p*
	n (%)	95% confidence interval	n (%)	95% confidence interval	
**2 weeks**	111 (96.5)	91.3–99.0	101 (87.8)	80.4–93.1	0.006
**4 weeks**	113 (98.3)	93.9–99.8	108 (93.9)	87.9–97.5	0.180
**16 weeks**	113 (98.3)	93.9–99.8	110 (95.7)	90.1–98.6	0.453

Note: *p* , descriptive level of the McNemar test.

**Fig. 2:**
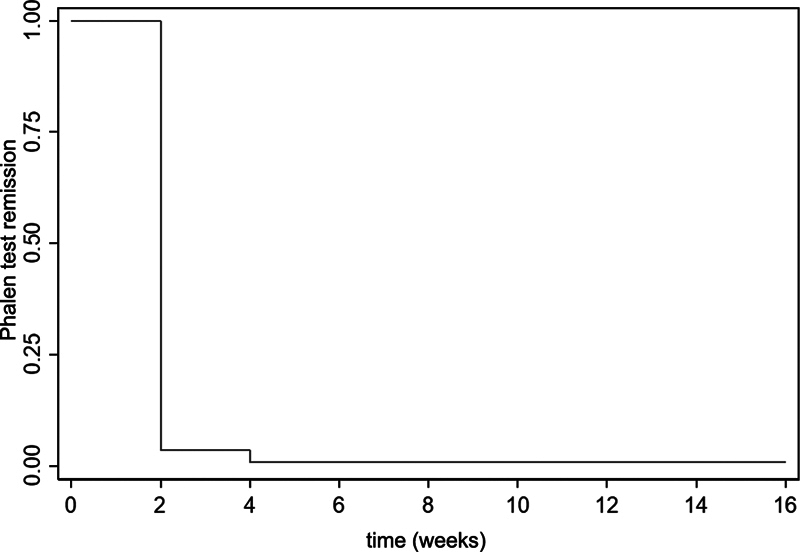
Kaplan-Meier curve showing Phalen test remission.

**Fig. 3:**
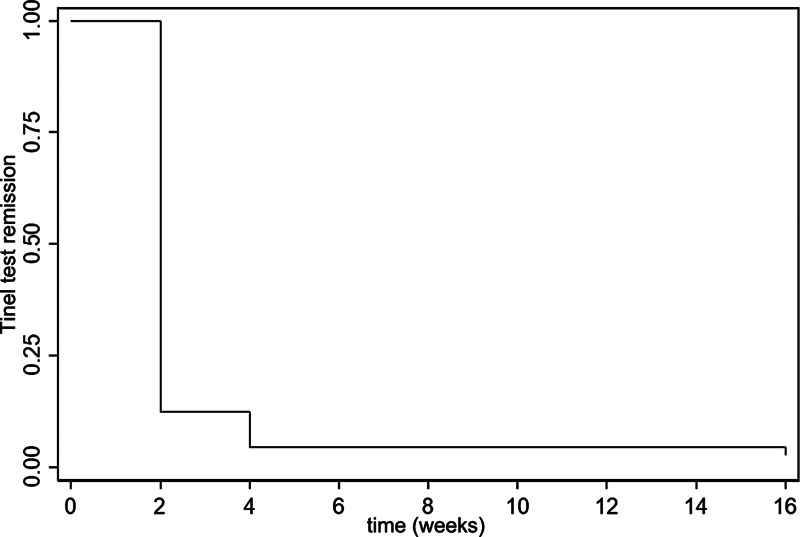
Kaplan-Meier curve showing remission of the Tinel sign.

Fig. 2 and 3 show the Kaplan-Meier curve with the estimated probability of the time until remission and the mean time until remission of the Phalen test and the Tinel sign respectively.

Table 2 shows the estimated probability of the time until remission of the Phalen test 2, 4, and 16 weeks postoperatively, which were of 3.54% (95% confidence interval [95%CI]: 1.16%–8.17%), 0.88% (95%CI: 0.08%–4.38%) and 0.88% (95%CI: 0.08%–4.38%) respectively. The probability estimates of the time until remission of the Tinel sign 2, 4, and 16 weeks postoperatively were o 12.39% (95%CI: 7.13%–19.18%), 4.42% (95%CI %: 1.65%–9.36%) and 2.65% (95%CI: 0.70%–6.94%) respectively. Two patients who did not present a positive Phalen test and another two who did not present a positive Tinel sign during the evaluation of the clinical diagnosis of CTS through the CTS-6 were excluded from this calculation. 7

**Table 2:** Results of the analysis of the Kaplan-Meier curves considering the time until test remission as the primary outcome (n = 113)

	Accumulated percentage of test remission		
	2 weeks postoperatively	4 weeks postoperatively	16 weeks postoperatively
**Phalen test**	3.54 ± 1.74	0.88 ± 0.88	0.88 ± 0.88
**Tinel sign**	12.39 ± 3.10	4.42 ± 1.93	2.65 ± 1.51

Note: ± Standard error of the mean.

Table 3 shows that there was no remission of the Phalen test in 4 patients (3.5%), neither of the Tinel sign in 14 patients (12.2%) 2 weeks postoperatively. At the end of the 16-week postoperative clinical course, there was no remission of the Phalen test in 2 patients (1.7%), netiher of the Tinel sign in 5 patients (4.3%).

**Table 3:** Results of the Phalen test and Tinel sign at each point in the postoperative (PO) evaluation (n = 115)

	Phalen test		Tinel sign	
	n	%	n	%
**2 weeks PO**	**115**	**100.0%**	**115**	**100.0%**
No remission	4	3.5%	14	12.2%
Remission	111	96.5%	101	87.8%
**4 weeks PO**	**115**	**100.0%**	**115**	**100.0%**
No remission	2	1.7%	7	6.1%
Remission	113	98.3%	108	93.9%
**16 weeks PO**	**115**	**100.0%**	**115**	**100.0%**
No remission	2	1.7%	5	4.3%
Remission	113	98.3%	110	95.7%

Table 4 shows that there was a reduction of 1.8 points on average in the score on the symptom severity scale, as well as a reduction of 1.6 points on average in the score on the functional status scale, after the surgical treatment compared to the preoperative clinical assessment ( *p* < 0.001).

**Table 4:** Comparison of the pre- and postoperative scores on the Boston Carpal Tunnel Questionnaire

Boston Carpal Tunnel Questionnaire	Postoperative: mean ± standard deviation	Preoperative: mean ± standard deviation	Post- and Preoperative difference: mean ± standard deviation	*p*
Symptom severity scale	1.7 (0.7)	3.5 (0.7)	−1.8 (0.9)	< 0.001
Functional status scale	2.0 (1.0)	3.6 (0.9)	1.6 (1.1)	< 0.001

Notes: Kolmogorov-Smirnov test for the symptom severity score: clinical assessment (p = 0.709); Kolmogorov-Smirnov test for the functional status score: clinical evaluation (p = 0.728).

## Discussion

When exploring prognostic factors regarding the conservative and surgical treatment for CTS, most studies in the literature are retrospective and with small samples, or they are carried out in the context of randomized clinical trials, which have limitations. 6 There are few prospective studies that address prognostic factors related to clinical test remission in CTS patients treated surgically, which enhances the value of the design of the current study, a cohort study on prognosis. 13 14 In the present study, the remission of the Phalen test and Tinel sign after the second postoperative week was statistically significant for most patients, showing that the these clinical tests are favorable prognostic factors for the postoperative clinical course. Gong et al. 15 (2008) concluded that the Phalen tests and Tinel sign were not statistically significant prognostic factors in the evaluation of patients treated surgically for CTS. Fakhouri et al. 16 (2017) carried out an observational and prospective study on 620 CTS patients undergoing surgical treatment, and. they concluded that the Phalen test had a better impact on the results than the Tinel sign after 2 postoperative weeks. Most patients in whom the Phalen test was positive in the preoperative period presented remission of this test 2 weeks postoperatively, with a good response to the surgical treatment. The response to the surgical treatment was not considered good if the Phalen test was negative in the preoperative period and remained negative within 2 weeks and until 24 weeks postoperatively. 16 In the present stidy, we obtained similar results regarding the Phalen test, which is demonstrated by the incidence of remission of the Phalen test 2 weeks postoperatively, which was superior to that of the Tinel sign, and also by the average time until remission of the Phalen test, which was earlier than that of the Tinel sign. The response to the surgical treatment was evaluated by the results obtained with the reduction in BCTQ scores, with an average of 1.8 for the symptom severity scale and of 1.6 points for the functional status scale. 8 Aversano et al. 17 (2022) obtained a mean improvement in BCTQ scores of 1.38 ± 0.77 points for both scales in the postoperative period. In the present study, the reasons why the Phalen test and Tinel sign did not remit after 2 weeks and until the end of 16 postoperative weeks are variable; they may be related to the patient's advanced age, individual scarring factors, the regeneration process of nerve fibers that were still recovering, the presence of anatomical variations, the lack of remission of postoperative paresthesia, and CTS in advanced stages. 6 14 18 The limitations of the present study refer to a longer follow-up of the patients' postoperative clinical course and the evaluation of a single variable, showing the study's simplicity in providing little prognostic information. Future research shows that strategies to measure patient-centered outcomes are increasingly being used in prognostic studies, rather than valuing outcomes measured accurately or with technological resources at the expense of clinical relevance.

## Conclusions

The present study showed that the usefulness of the Phalen test and the Tinel sign goes beyond the clinical diagnosis of CTS. Remission of the Phalen test occurs earlier than that of the Tinel sign, but when both occur starting at the second postoperative week, they are favorable prognostic factors for the clinical course. The reduction in postoperative scores on the BCTQ proved the effectiveness of the surgical treatment, with improved health-related quality of life.
