# Supplementary material for: Usefulness of the Phalen Test and the Tinel Sign in the Prognosis and the Impact on Quality of Life of Patients with Carpal Tunnel Syndrome Undergoing Classical Open Carpal Tunnel Release*
Source: Rev Bras Ortop (Sao Paulo). 2024 Mar 21;59(1):e54–9. doi: 10.1055/s-0044-1779318 (PMC10957282; doi:10.1055/s-0044-1779318)
Supplement: Related texts

# Research article: Utilidade do teste de Phalen e do sinal de Tinel no prognóstico e o impacto na qualidade de vida de pacientes com síndrome do túnel do carpo submetidos a liberação aberta clássica do túnel do carpo*

**Keywords:** síndrome do túnel do carpo, fator prognóstico, qualidade de vida, curso clínico

## Abstract

**Objetivo:** Avaliar a utilidade do teste de Phalen e do sinal de Tinel no prognóstico e o impacto na qualidade de vida no curso clínico de pacientes com síndrome do túnel do carpo submetidos ao tratamento cirúrgico por via aberta clássica.

**Métodos:** Trata-se de um estudo de coorte sobre prognóstico. Foram incluídos 115 pacientes com alta probabilidade de diagnóstico clínico de síndrome do túnel do carpo com indicação de tratamento cirúrgico. Todos os pacientes foram submetidos ao teste de Phalen e ao sinal de Tinel, e responderam ao questionário de Boston antes e depois do tratamento cirúrgico.

**Resultados:** As estimativas de probabilidade do tempo até a remissão do teste de Phalen em 2, 4 e 16 semanas pós-operatórias foram de 3,54% (intervalo de confiança de 95% [IC95%]:1,16%–8,17%), 0,88% (IC95%: 0,08%–4,38%) e 0,88% (IC95%: 0,08%–4,38%), respectivamente, e, do sinal de Tinel, foram de 12,39% (IC95%: 7,13%–19,18%), 4,42% (IC95%: 1,65%–9,36%) e 2,65% (IC95%: 0,70%–6,94%), respectivamente. Na pontuação pós-operatória no Questionário de Boston, houve redução de 1,8 ponto para a gravidade dos sintomas ( *p* < 0,001), e de 1,6 ponto para o estado funcional ( *p* < 0,001).

**Conclusão:** A remissão do teste de Phalen foi mais precoce do que a do sinal de Tinel, mas, realizados a partir da segunda semana de evolução pós-operatória, esses testes foram fatores prognósticos favoráveis ao curso clínico, com melhora da qualidade de vida.

## Introdução

A síndrome do túnel do carpo (STC) é caracterizada por compressão do nervo mediano no punho. 1 Na prática clínica de rotina, utilizamos tradicionalmente os resultados do teste de Phalen e do sinal de Tinel como achados clínicos mais representativos no diagnóstico clínico da STC. 2 3 4 Entretanto, estes testes clínicos provocativos são eventos não percebidos pelos pacientes, mas podem ser explorados na avaliação do curso clínico de pacientes com STC, tanto no diagnóstico clínico quanto na análise do tratamento e no prognóstico. 5 6 Estudos de prognóstico com qualidade levam a uma melhor compreensão da progressão da doença e a uma melhor indicação de tratamentos eficazes, além de fornecerem informações mais confiáveis sobre o risco de um resultado negativo a ser comunicado aos pacientes. 6 Na condição de modelo de raciocínio clínico baseado em evidências, este estudo se propõe avaliar a utilidade do teste de Phalen e do sinal de Tinel no prognóstico e o impacto na qualidade de vida de pacientes com alta probabilidade de diagnóstico clínico de STC submetidos ao tratamento cirúrgico pela via aberta clássica.

## Materiais e Métodos

Trata-se de um estudo de coorte sobre prognóstico, primário, longitudinal, prospectivo, de centro único, aprovado pelo Comitê de Ética em Pesquisa de nossa instituição. Incluímos 115 pacientes, todas do gênero feminino, com idades entre 40 e 80 anos, que concordaram e assinaram o termo de consentimento livre e esclarecido. As pacientes haviam sido submetidas a tratamento conservador prévio sem melhora clínica efetiva e com indicação de tratamento cirúrgico, e apresentavam uma pontuação ≥ 12 na escala de 6 itens de sintomas de STC (Six-item CTS Symptoms Scale, CTS-6, em inglês), que indica a probabilidade de diagnóstico clínico de STC. 7 Os 6 critérios diagnósticos utilizados por este instrumento e suas respectivas pontuações são: parestesia (3,5), parestesia noturna (4,0), hipotrofia e ou atrofia da musculatura tenar (5,0), sinal de Tinel (4,0), teste de Phalen (5,0) e teste estático da discriminação dos 2 pontos (5,0). 7 Excluímos pacientes com radiculopatia cervical, outras síndromes compressivas dos membros superiores, polineuropatia, histórico de liberação cirúrgica do túnel do carpo e sequelas de fratura do punho. Recrutamos 10% a mais do que o total de pacientes elegíveis para cobrir possíveis perdas ou exclusões no decorrer do estudo. Todos os pacientes elegíveis responderam ao Questionário de Boston (QB), no período pré-operatório e ao final do seguimento; trata-se de um questionário específico para STC traduzido e validado para a língua portuguesa, autoaplicado, e que avalia uma escala de gravidade dos sintomas e outra de estado funcional. 8 Como prática clínica de rotina, os pacientes foram submetidos a ultrassonografia (US) do punho e eletroneuromiografia (ENMG). Em seguida, os pacientes foram submetidos ao tratamento cirúrgico, realizado em caráter ambulatorial no centro cirúrgico do hospital de nossa instituição de forma consecutiva e por um mesmo cirurgião da mão. A técnica utilizada foi a liberação por via aberta clássica do túnel do carpo. 9 Após o tratamento cirúrgico, os pacientes receberam seguimento pós-operatório ambulatorial por 16 semanas. O teste de Phalen e o sinal de Tinel foram realizados uma vez como parte do diagnóstico clínico da STC no pré-operatório e com 2, 4 e 16 semanas de pós-operatório. No teste de Phalen, o paciente fica apoiado na mesa de exame com o cotovelo posicionado em 90° de flexão e com o punho posicionado em flexão passiva máxima da mão acometida. 10 11 O aparecimento de parestesia no território de distribuição do nervo mediano na mão entre 30 e 60 segundos indica o resultado positivo. No sinal de Tinel, realiza-se uma digito percussão sobre a região da prega de flexão distal do punho no trajeto do nervo mediano da mão acometida. 11 A sensação de “choque” ou incômodo no local da percussão ou irradiada para o território de distribuição do nervo mediano na mão acometida indica o resultado positivo. A Fig. 1 mostra de forma resumida cada etapa deste estudo.

**Fig. 1:** Fluxograma do estudo. Abreviaturas: CTS-6, Instrumento de probabilidade de diagnóstico clínico para síndrome do túnel do carpo; 7 BCTQ, Questionário de Boston. 8

Como desfecho primário deste estudo, as estimativas da probabilidade do tempo até a remissão em cada momento de avaliação e do tempo médio até a remissão em cada teste clínico foram feitas pela análise da curva de Kaplan-Meier. Como desfecho secundário, as mudanças na pontuação no QB foram estimadas por meio da análise de variância ( *analysis of variance* , ANOVA, em inglês) e do teste de Kolmogorov-Smirnov. Para todos os testes estatísticos, adotou-se um nível de significância de 5%. As análises estatísticas foram realizadas utilizando-se os programas IBM SPSS Statistics for Windows (IBM Corp., Armonk, NY, Estados Unidos), versão 20.0, e Stata (StataCorp LLC, College Station, TX, Estados Unidos), versão 17. 12

## Resultados

A média da idade das 115 pacientes foi de 52,9 (desvio padrão [DP] = ± 9,1) anos, com idade mínima de 40 anos e máxima de 79 anos. Com relação ao tempo da doença, a média observada foi de 4 (DP = ± 3,2) anos, com um mínimo de 1 ano e máximo de 20 anos. A mediana foi de 3 anos de tempo de doença. Em 5 pacientes (4,3%), houve complicações pós-operatórias, sendo que 1 paciente evoluiu com síndrome dolorosa regional complexa, outro apresentou deiscência da cicatriz cirúrgica devido a uma infecção superficial, 1 evoluiu com cicatriz hipertrófica e dolorosa, e outros 2 pacientes evoluíram com dor no pilar ósseo.

A Tabela 1 mostra que a incidência da remissão do teste de Phalen em 2 semanas pós-operatório foi superior à do sinal de Tinel ( *p* = 0,006). Não se verificaram diferenças na incidência da remissão com relação aos demais momentos de avaliação dos dois testes clínicos.

**Tabela 1:** Incidências da remissão por tipo de teste e momentos de avaliação

Período pós-operatório	Teste de Phalen		Sinal de Tinel		*p*
	n (%)	Intervalo de confiança de 95%	n (%)	Intervalo de confiança de 95%	
**2 semanas**	111 (96,5)	91,3 a 99,0	101 (87,8)	80,4 a 93,1	0,006
**4 semanas**	113 (98,3)	93,9 a 99,8	108 (93,9)	87,9 a 97,5	0,180
**16 semanas**	113 (98,3)	93,9 a 99,8	110 (95,7)	90,1 a 98,6	0,453

Nota: *p* , nível descritivo do teste de McNemar.

As Figs. 2 e 3 mostram a curva de Kaplan-Meier com a estimativa de probabilidade do tempo até a remissão e do tempo médio até a remissão do teste de Phalen e do sinal de Tinel, respectivamente.

**Fig. 2:** Curva de Kaplan-Meier mostrando a remissão do teste de Phalen. Estimativa do tempo médio até a remissão do teste de Phalen = 2,18 semanas; intervalo de confiança de 95% (IC95%): 1,93–2,43.

**Fig. 3:** Curva de Kaplan-Meier mostrando a remissão do sinal de Tinel. Estimativa do tempo médio até a remissão do sinal de Tinel = 2,78 semanas; IC95%: 2,24–3,32.

A Tabela 2 mostra a estimativa de probabilidade do tempo até remissão do teste de Phalen em 2, 4 e 16 semanas pós-operatórias, que foram de 3,54% (intervalo de confiança de 95% [IC95%]: 1,16%–8,17%), 0,88% (IC95%: 0,08%–4,38%) e 0,88% (IC95%: 0,08%–4,38%), respectivamente. Já as estimativas de probabilidade do tempo até remissão do sinal de Tinel em 2, 4 e 16 semanas pós-operatórias foram de 12,39% (IC95%: 7,13%–19,18%), 4,42% (IC95%: 1,65%–9,36%) e 2,65% (IC95%: 0,70%–6,94%), respectivamente. Foram excluídos deste cálculo duas pacientes que não apresentaram o teste de Phalen positivo e outras duas que não apresentaram o sinal de Tinel positivo durante a avaliação do diagnóstico clínico de STC pela CTS-6. 7

**Tabela 2:** Resultados da análise das curvas de Kaplan-Meier considerando como desfecho primário o tempo até a remissão do teste (n = 113)

	Porcentagem acumulada de remissão do teste		
	2 semanas de pós-operatório	4 semanas de pós-operatório	16 semanas de pós-operatório
**Teste de Phalen**	3,54 ± 1,74	0,88 ± 0,88	0,88 ± 0,88
**Sinal de Tinel**	12,39 ± 3,10	4,42 ± 1,93	2,65 ± 1,51

Nota: Média ± erro padrão.

A Tabela 3 mostra que, com 2 semanas de curso clínico pós-operatório, não houve remissão do teste de Phalen em 4 pacientes (3,5%), nem do sinal de Tinel em 14 pacientes (12,2%). Ao final de 16 semanas, não houve remissão do teste de Phalen em 2 pacientes (1,7%) e do sinal de Tinel em 5 pacientes (4,3%).

**Tabela 3:** Resultados do teste de Phalen e do sinal de Tinel em cada momento da avaliação pós-operatória (PO) (n = 115)

	Teste de Phalen		Sinal de Tinel	
	n	%	n	%
**2 semanas PO**	**115**	**100,0%**	**115**	**100,0%**
Sem remissão	4	3,5%	14	12,2%
Remissão	111	96,5%	101	87,8%
**4 semanas PO**	**115**	**100,0%**	**115**	**100,0%**
Sem remissão	2	1,7%	7	6,1%
Remissão	113	98,3%	108	93,9%
**16 semanas PO**	**115**	**100,0%**	**115**	**100,0%**
Sem remissão	2	1,7%	5	4,3%
Remissão	113	98,3%	110	95,7%

A Tabela 4 mostra que houve uma redução de 1,8 pontos em média na pontuação na escala de gravidade dos sintomas, e uma redução de 1,6 pontos em média na pontuação na escala do estado funcional, após o tratamento cirúrgico em comparação com a avaliação clínica pré-operatória ( *p* < 0,001).

**Tabela 4:** Comparação das pontuações no Questionário de Boston no pré e pós-operatório

Questionário de Boston	Pós-operatório: média(± desvio padrão)	Pré-operatório: média(± desvio padrão)	Diferença entre pós e pré-operatório: média(± desvio padrão)	*p*
Escala de gravidade dos sintomas	1,7(± 0,7)	3,5(± 0,7)	−1,8(± 0,9)	< 0,001
Escala de estado funcional	2,0(± 1,0)	3,6(± 0,9)	1,6(± 1,1)	< 0,001

Notas: Teste de Kolmogorov-Smirnov para escore de gravidade dos sintomas: avaliação clínica ( *p* = 0,709); teste de Kolmogorov-Smirnov para escore de estado funcional: avaliação clínica ( *p* = 0,728).

## Discussão

A maioria dos estudos na literatura, quando exploram fatores prognósticos no tratamento conservador e cirúrgico na STC, são retrospectivos e com amostras pequenas, ou realizados no contexto de ensaios clínicos randomizados, que apresentam limitações. 6 São escassos os estudos prospectivos que abordam fatores prognósticos relacionados à remissão de testes clínicos em pacientes com STC tratados cirurgicamente, o que valoriza o desenho do estudo atual, de coorte sobre prognóstico. 13 14 Neste estudo, a remissão do teste de Phalen e do sinal de Tinel após a segunda semana pós-operatória foi estatisticamente significativa para a maioria dos pacientes, o que mostra que estes testes clínicos são fatores prognósticos favoráveis ao curso clínico pós-operatório. Gong et al. 15 (2008) concluíram que o teste de Phalen e o sinal de Tinel não foram fatores prognósticos estatisticamente significativos na avaliação de pacientes com STC tratados cirurgicamente. Fakhouri et al. 16 (2017) realizaram um estudo observacional e prospectivo em 620 pacientes submetidos ao tratamento cirúrgico, e concluíram que o teste de Phalen apresentou melhor impacto nos resultados do que o sinal de Tinel após 2 semanas de pós-operatório. A maioria dos pacientes em que o teste de Phalen foi positivo no pré-operatório apresentaram remissão deste teste após 2 semanas pós-operatórias, com boa resposta ao tratamento cirúrgico. A resposta ao tratamento cirúrgico não foi considerada boa se o teste de Phalen foi negativo no pré-operatório e continuou negativo em 2 semanas e até que se completassem 24 semanas pós-operatórias. 16 Obtivemos resultados semelhantes em relação ao teste de Phalen neste estudo, demonstrados pela incidência de remissão em 2 semanas pós-operatórias, que foi superior à do sinal de Tinel, e também pelo tempo médio até a remissão do teste de Phalen, que foi mais precoce do que o do sinal de Tinel. Já a resposta ao tratamento cirúrgico foi avaliada pelos resultados obtidos com a redução das pontuações no QB, sendo em média de 1,8 para a escala de gravidade dos sintomas, e de 1,6 pontos para a escala de estado funcional. 8 Aversano et al. 17 (2022) obtiveram uma melhora média das pontuações no QB de 1,38 ± 0,77 pontos para as 2 escalas no pós-operatório. No presente estudo, os motivos pelos quais não ocorreu a remissão do teste de Phalen e do sinal de Tinel após 2 semanas e até o final de 16 semanas de pós-operatório são variáveis; podem estar relacionados à idade avançada do paciente, a fatores cicatriciais individuais, ao processo de regeneração das fibras nervosas que ainda estavam em recuperação, à presença de variações anatômicas, à ausência da remissão da parestesia pós-operatória e aos estágios avançados da STC. 6 14 18 As limitações deste estudo referem-se a um tempo maior de seguimento dos pacientes e à avaliação de apenas uma variável, o que mostra uma simplicidade do estudo em fornecer poucas informações do prognóstico. Pesquisas futuras mostram que cada vez mais, estratégias para medir desfechos centrados no paciente vêm sendo utilizados em estudos de prognóstico, ao invés de valorizar desfechos medidos com precisão ou com recursos tecnológicos às custas da relevância clínica.

## Conclusão

Este estudo mostrou que a utilidade do teste de Phalen e do sinal de Tinel vai além do diagnóstico clínico da STC. A remissão do teste de Phalen é mais precoce do que a do sinal de Tinel, mas, realizados a partir da segunda semana de evolução pós-operatória, esses testes são fatores prognósticos favoráveis ao curso clínico. A redução nas pontuações pós-operatórias no QB comprovaram a eficácia do tratamento cirúrgico, com melhora da qualidade de vida relacionada à saúde.
